# β-Actin: Not a Suitable Internal Control of Hepatic Fibrosis Caused by *Schistosoma japonicum*

**DOI:** 10.3389/fmicb.2019.00066

**Published:** 2019-01-31

**Authors:** Beibei Zhang, Xiaoying Wu, Jiahua Liu, Langui Song, Qiuyue Song, Lifu Wang, Dongjuan Yuan, Zhongdao Wu

**Affiliations:** ^1^Department of Parasitology, Zhongshan School of Medicine, Sun Yat-sen University, Guangzhou, China; ^2^Key Laboratory of Tropical Disease Control (SYSU), Ministry of Education, Sun Yat-sen University, Guangzhou, China; ^3^Provincial Engineering Technology Research Center for Biological Vector Control, Sun Yat-sen University, Guangzhou, China; ^4^School of Public Health, Fudan University, Shanghai, China; ^5^College of Veterinary Medicine, South China Agricultural University, Guangzhou, China

**Keywords:** β-actin, schistosomiasis japonica, hepatic fibrosis, housekeeping gene, internal control

## Abstract

Schistosomiasis japonica is a significant health problem that leads to morbidity and mortality of humans. It is characterized by hepatic granulomatous response and fibrosis caused by eggs deposition in the liver. β-actin, a traditional housekeeping gene, is widely used as an internal control to normalize gene and protein expression. However, β-actin expression can fluctuate upon the treatment with pharmacological agents or under some physiological and pathological conditions. In this study, we found that the expressions of both β-actin mRNA and protein increased significantly with hepatic fibrosis formation after 6 weeks infection with *Schistosoma japonicum* and kept high level during the progression of hepatic fibrosis, while the levels of β-Tubulin and glyceraldehyde-3-phosphate dehydrogenase (GAPDH) remained stable. The dynamic change of β-actin was similar with the profibrogenic factors, including α-SMA, Collagen I, and Collagen III. We employed immunofluorescence staining and further showed that the expression level of β-actin was positively correlated with α-SMA. What is more, there was a positive correlation between the level of β-actin mRNA and the content of hydroxyproline in liver. This study provides evidences that β-actin is variable and unsatisfied for application as an internal control in hepatic fibrosis induced by *S. japonicum* infection.

## Introduction

Schistosomiasis japonica, caused by *Schistosoma japonicum* infection, continues to be a significant health problem that leads to human morbidity and mortality, especially in lake and marshland regions of China, the Philippines, and Sri Lanka, as well as the area near Lindu Lake (Barsoum et al., 2013; Colley et al., 2014). *S. japonicum* is one of the three main *Schistosoma* species that affect humans, comprising *Schistosoma mansoni*, *S. japonicum*, and *Schistosoma haematobium.* In schistosomasis, eggs are released by adult worms and deposit in the presinusoidal capillary venules of the liver. Granulomous formation accompanies with recruitment of inflammatory cells, including macrophages, eosinophils, and activated hepatic stellate cells (HSCs) around the eggs. These granulomas contribute to severe fibrosis and even give rise to portal hypertension and portacaval shunting due to the disturbance of the hepatic blood supply (Chuah et al., 2014; Colley et al., 2014; Carson et al., 2018). Although great achievements have been attained in the control of schistosomiasis japonica in China, there were still 54,454 schistosomiasis patients in China at the end of 2016, and 30,573 of those patients were documented as advanced schistosomiasis (Li-Juan et al., 2017). Newly developed cases can also be reported in endemic regions (Song et al., 2017). Thus, it is still a major parasitic disease that needs sensitive diagnose and efficient therapy that prevents and targets fibrosis and portal hypertension. By now, a lot of researches have been conducted for clarifying the pathogenesis and prevention of schistosomiasis japonica.

β-actin is widely used as an internal control in a variety of researches on liver diseases because it is regarded as a highly stable housekeeping gene (Sturzenbaum and Kille, 2001; Ruan and Lai, 2007). It is one isoform in the actin family, functions as a cytoskeleton protein that forms filaments. This family includes β_cyto_-actin, γ_cyto_-actin, α_skeletal_-actin, α_cardiac_-actin, α_smooth_-actin, and γ_smooth_-actin. Among them, β_cyto_-actin and γ_cyto_-actin are the major components of cytoskeleton proteins. α_skeletal_-actin, α_cardiac_-actin, α_smooth_-actin, and γ_smooth_-actin are the main components of muscle fiber and are primarily expressed in skeletal striated muscles, smooth muscles, intestinal muscles, and cardiac muscles, respectively. Different isoforms possess some unique and overlapping cellular functions (MacQueen et al., 2005). α-Smooth muscle actin (α-SMA) is a hallmark of activated myofibroblasts and has been extensively used to indicate the occurrence and severity of fibrosis in liver diseases (Akpolat et al., 2005; Thomas et al., 2011; Kuramitsu et al., 2013). In hepatic fibrosis associated with schistosomiasis, the expression level of α-SMA is elevated (Carson et al., 2018). β-actin shares more than 93% sequence identity with α-SMA. But it remains unknown whether the expression of β-actin keeps stable through the progression of hepatic fibrosis in schistosomiasis japonica.

In this study, we explored the expression pattern of hepatic β-actin at both the mRNA and protein levels at different time points during *S. japonicum* infection in mice. We confirmed that the β-actin level increased significantly during the progression of hepatic fibrosis induced by *S. japonicum* infection. Additionally, there was a positive correlation between the level of hepatic β-actin mRNA and the severity of hepatic fibrosis. Our data highlights that β-actin is unsuitable for application as an internal control of hepatic fibrosis in schistosomiasis japonica.

## Materials and Methods

### Ethics Statement

All animal experiments were performed in strict accordance with the Guide for the Care and Use of Laboratory Animals of the National Institutes of Health. The protocol was approved by the Sun Yat-sen University Committee for Animal Research (No. 2016-104).

### Animals and Parasites

Male BALB/c mice (6 weeks old) were purchased from the Experimental Animal Center of Guangdong Province, and *Oncomelania hupehensis* infected with *S. japonicum* cercariae were obtained from National Institute of Parasitic Diseases, Chinese Center for Disease Control and Prevention in Shanghai. The mice were exposed percutaneously with 15 ± 2 cercariae of *S. japonicum* originating from *O. hupehensis* snails as previously described (Peng et al., 2016). Six mice were distributed in each group. Mice were sacrificed under deep anesthesia. Livers were collected at 4, 6, 8, 10, and 14 weeks post infection (wpi).

### CCl4 Animal Experiments

Male BALB/c mice (6 weeks old) weighing 21–25 g were obtained from the Experimental Animal Center of Guangdong Province. For induction of fibrotic liver, mice were intraperitoneally injected with 1.6 g/kg body weight of CCl4 (mixed with olive oil) twice 1 week for 8 weeks. Animals were sacrificed under deep anesthesia. Livers were collected and snap frozen in liquid nitrogen for western blot detection.

### Histopathology and Immunofluorescence Detection

The livers were collected from the infected mice and fixed in 4% paraformaldehyde. Then, the tissues were embedded in paraffin and sectioned. The sizes of hepatic granulomas were assessed by H&E staining, and the deposited of collagen was detected by masson’s trichrome staining. All these specimens were visualized under an optical microscope (Olympus, Tokyo, Japan).

Frozen liver sections with a thickness of 6 μm were prepared for immunofluorescence analysis. The sections were fixed in 4% paraformaldehyde for 20 min and undergone gradient dehydration with ethanol. After washing with PBS three times for 5 min each; 1% bovine serum albumin was used to block the sections. The sections were incubated with primary antibodies mouse anti-β-actin (58169, Cell Signaling Technology, Boston, MA, United States) and rabbit anti-α-SMA (19245, Cell Signaling Technology, Boston, MA, United States) at 4°C overnight for dual staining. The antibody β-actin did not cross-react with α-SMA. The dilution of both primary antibodies is 1:500. After washing with PBS three times, the corresponding secondary antibodies Alexa Fluor 594-conjugated goat anti-mouse IgG (150113, Cell Signaling Technology, Boston, MA, United States) and Alexa Fluor 488-conjugated donkey anti-rabbit IgG (150076, Cell Signaling Technology, Boston, MA, United States) were diluted at 1:200 and applied for 2-h incubation at room temperature. After washing, all sections were counterstained with DAPI (Beyotime Biotechnology, Shanghai, China) for 3 min. Pictures were captured with the same exposure time by Olympus BX-63 fluorescence microscopy (Olympus, Tokyo, Japan). The integrated optical density (IOD) of β-actin and α-SMA were analyzed by Image Pro Plus 6.0 software.

### Western Blotting

Livers were sampled from *S. japonicum* infected mice at each time point and from mice stimulated with CCl4. Total liver proteins were extracted from liver tissues (30–50 mg) with ice-cold RIPA lysis buffer (Beyotime Biotechnology, Shanghai, China) containing complete protease inhibitor and PhosSTOP phosphatase inhibitor (both from Roche, Mannheim, Germany). After centrifuging with 10,000 *g* for 10 min, the supernatant was removed to another tube. Then BCA protein assay kit (Beyotime Biotechnology, Shanghai, China) was used to determine the concentration of total protein. Equal amounts (60 μg) of protein were loaded and separated on a 10% polyacrylamide gel with 200 V for 2 h and then transferred to a 0.22 μm PVDF membrane (Millipore, MA, United States) with 300 mA for 3 h. After blocking in PBS containing 5% milk and 0.1% Tween-20 for 2 h at room temperature, the membranes were incubated with primary antibodies mouse anti-β-actin (58169, Cell Signaling Technology, Boston, MA, United States, used at 1:2000 dilution), mouse anti-glyceraldehyde-3-phosphate dehydrogenase (GAPDH) (G8795, Sigma-Aldrich, St. Louis, MO, United States, used at 1:2000 dilution), and mouse anti-β-Tubulin (T0198, Sigma-Aldrich, St. Louis, MO, United States, used at 1:2000 dilution), rabbit anti-α-SMA (19245, Cell Signaling Technology, Boston, MA, United States, used at 1:1000 dilution), rabbit anti-Collagen I (COL1A1) (BA0325, Boster, Wuhan, China, used at 1:1000 dilution), rabbit anti-Collagen III (COL3A1) (BM1625, Boster, Wuhan, China, used at 1:1000 dilution) at 4°C overnight. Specially, mouse anti-β-actin does not cross-react with α-SMA. To verify the expression of β-actin, another two primary antibodies mouse anti-β-actin were also used (60008, Proteintech, Wuhan, China; Beijing Ray Antibody Biotech, Beijing, China, used at 1:2000 dilution). After washing 5 min with three times, membranes were incubated with the appropriate anti-mouse IgG (7076S, Santa Cruz, CA, United States, used at 1:5000 dilution) or anti-rabbit IgG (7074S, Santa Cruz, CA, United States, used at 1:5000 dilution). Protein bands were visualized using chemiluminescent HRP substrate (Millipore, MA, United States) and an Amersham Imager 600 system (GE, CT, United States). Image J2x software was used to quantify the density of each band by densitometric analysis. The relative expression of each protein was normalized to GAPDH. Fold change was calculated by comparing to the control group.

### RNA Extraction and Real-Time qPCR

Total RNA was isolated from around 30 mg liver tissue using TRIzol Reagent (Invitrogen, New York, NY, United States) according to the manufacturer’s protocol. The quality and quantity of RNA were assessed by NanoDrop 2000 spectrophotometer (Thermo Fisher, Waltham, MA, United States). Then 3 μg was used to reverse transcribed to cDNA using the RevertAid First Strand cDNA Synthesis Kit (Thermo Fisher, Waltham, MA, United States). The expression levels of α-SMA, Collagen I, and Collagen III were determined using SYBR Premix Ex Taq^TM^ (TaKaRa, Osaka, Japan) and CFX 96 touch instrument (Bio-Rad, CA, United States). The reaction procedure was as following: 95°C for 30 s; 40 cycles of 95°C for 5 s and 60°C for 30 s; 95°C for 10 s, 60°C for 10 s, 70°C for 10 s. GAPDH was used as an internal control, and the fold changes were quantified by the 2^-ΔΔCt^ method. The sequences of the primers are shown in Table 1 and all primer sequences were blasted in NCBI for ensuring their specificities.

**Table 1:** Primers used for mRNA analysis.

Genes	Primer	Sequence(5′–3′)
*β-actin*	Forward primer	TGGAATCCTGTGGCATCCATGAAAC
	Reverse primer	TAAAACGCAGCTCAGTAACAGTCCG
*GAPDH*	Forward primer	ACTCCACTCACGGCAAATTC
	Reverse primer	TCTCCATGGTGGTGAAGACA
*Tubulin*	Forward primer	AGGCCGGTGCTGAGTATGTCGT
	Reverse primer	TCACAAACATGGGGGCATCGGC
*α-SMA*	Forward primer	CACAGCCCTGGTGTGCGACAAT
	Reverse primer	TTGCTCTGGGCTTCATCCCCCA
*Collagen I*	Forward primer	TCCTGCGCCTAATGTCCACCGA
	Reverse primer	AAGCGACTGTTGCCTTCGCCTC
*Collagen III*	Forward primer	TCCTGGTGGCAAGGGTGATCGT
	Reverse primer	TGGAGCACCAGAAGGACCAGCA

For absolute real-time qPCR, the complete CDS of β-actin was amplified by PCR using total cDNA as a template and cloned into the pMD18-T vector (TaKaRa, Osaka, Japan) according to the manufacture’s instruction. The cloned sequence was confirmed by sequencing (Sangon Biotech, Shanghai, China). The concentration of the plasmid was then measured using NanoDrop 2000 spectrophotometer. The copy number of the plasmid was estimated, and a series of dilutions were prepared as standards. The copy numbers of β-actin in all samples were calculated using the standard curve.

### Determination of Hydroxyproline Content

Hydroxyproline is a significant component of collagen. For detection the content of hydroxyproline in liver, approximately 40 mg tissues were collected from each infected mouse. A commercially hydroxyproline assay kit was acquired from Jiancheng Institute of Biotechnology (A030-2, Jiancheng Institute of Biotechnology, Nanjing, China). All the procedure was strictly in accordance with the manufacturer’s instructions. The optical density (OD) of each sample was obtained at 550 nm using an ELISA reader (Sunrise, TECAN, Männedorf, Austria).

### Statistical Analysis

Data are expressed as the mean ± SEM. Statistical analyses were performed with SPSS 14.0. Statistical differences between groups were analyzed by independent-sample *t*-test. For comparing infected groups with control group, one-way analysis of variance (ANOVA) followed by Dunnett test or non-parametric test followed by Kruskal–Wallis *H*-test was used. Because the data did not display a normal distribution, Spearman’s correlation analysis was used to analyze the correlation between the concentration of hydroxyproline content and the level of β-actin mRNA or the correlation between the IOD of β-actin and α-SMA. Differences with *p* < 0.05 were deemed to be significant.

## Results

### The Expression Profile of Hepatic β-Actin in *S. japonicum* Infected Mice

**FIGURE 1:**
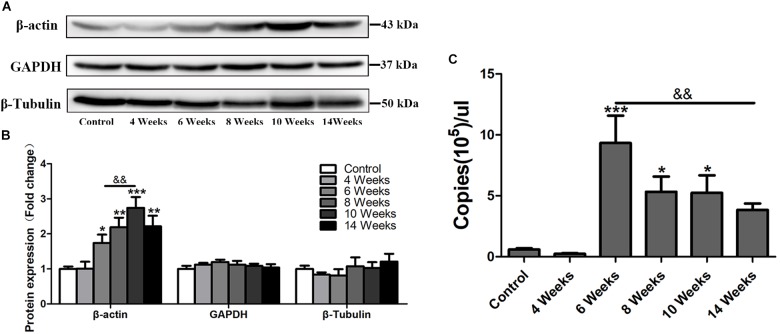
The expression profile of hepatic β-actin in *S. japonicum* infected mice. **(A)** Total liver lysates were subjected to detect the expression levels of β-actin, GAPDH, and β-Tubulin by western blot. **(B)** Densitometric analysis of β-actin, GAPDH, and β-Tubulin using Image J2x software. The fold changes were calculated by comparing to the control group. **(C)** Absolute quantification of β-actin by real-time quantitative PCR. Data represent the mean ± SE. ^∗^*P* < 0.05, infected groups vs control group; ^∗∗^*P* < 0.01, infected groups vs control group; ^∗∗∗^*P* < 0.001, infected groups vs control group. ^&&^*P* < 0.01.

β-actin is widely used as an internal control in molecular biological studies (Ruan and Lai, 2007). However, in this study, in the progress of hepatic fibrosis in *S. japonicum* infected mice, the protein level of β-actin was increased in the liver after 6 wpi (*p* < 0.05) and peaked at 10 wpi (*p* < 0.001), but decreased at 14 wpi (Figure 1A,B). Other two traditional internal controls GAPDH and β-Tubulin remained stable expression in the mouse liver after *S. japonicum* infection (Figure 1A,B). Immunofluorescent staining showed that the expression level of β-actin remained at the basal level at 4 wpi but dramatically increased after 6 wpi compared to the expression of the control group (Figure 4). It alsodecreased in 14 wpi. The mRNA level of β-actin was further detected by absolute quantification qPCR. As shown in Figure 1C, the β-actin mRNA levels at 6, 8, and 10 wpi were significantly higher than those in the control and *S. japonicum* infected mice at 4 wpi. Thus, hepatic β-actin expression was significantly changed in mice with *S. japonicum* infection, suggesting that β-actin might be an unsuitable internal control in schistosomiasis japonica.

### The Characteristic Hepatic Fibrosis in the Livers of *S. japonicum* Infected Mice

**FIGURE 2:**
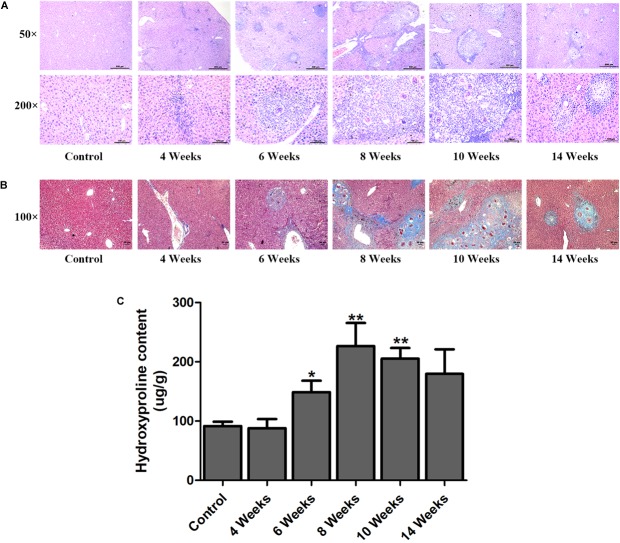
Determination of hepatic fibrosis in *S. japonicum* infected mice. Representative HE images **(A)** and Masson’s trichrome images **(B)** of hepatic fibrosis. **(C)** The concentration of hydroxyproline in liver was determined by a commercially hydroxyproline assay kit. Data are expressed as mean ± SE. ^∗^*P* < 0.05, infected groups vs control group; ^∗∗^*P* < 0.01, infected groups vs control group.

**FIGURE 3:**
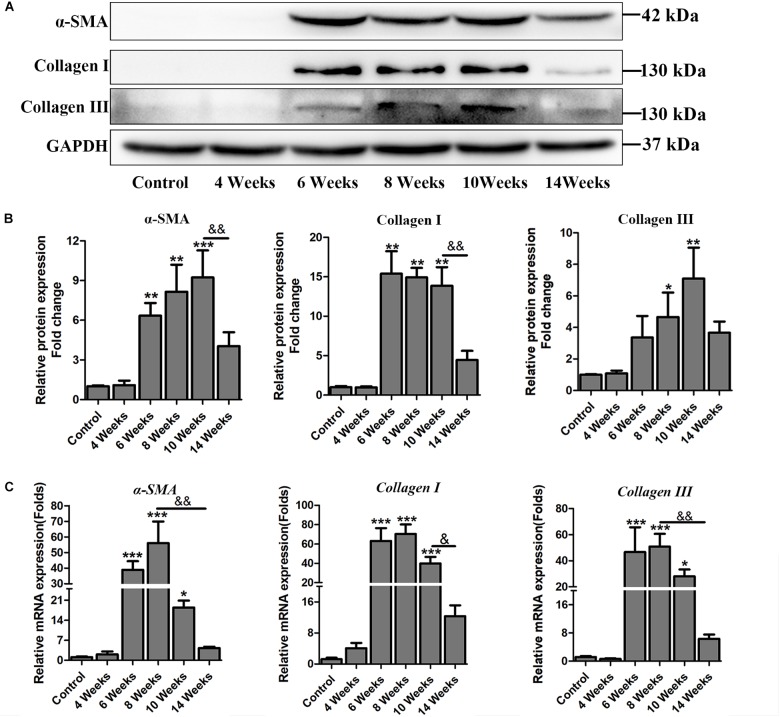
The expression profile of profibrogenic factors α-SMA, Collagen I, Collagen III in *S. japonicum* infected mice. **(A)** The protein levels of α-SMA, Collagen I, and Collagen III were detected by western blot and **(B)** quantified and normalized to GAPDH. The fold change was calculated by comparing to the control group. **(C)** The genes of *α-SMA*, *Collagen I*, and *Collagen III* were determined by qPCR. Data are expressed as mean ± SE. ^∗^*P* < 0.05, infected groups vs control group; ^∗∗^*P* < 0.01, infected groups vs control group; ^∗∗∗^*P* < 0.001, infected groups vs control group. ^&^*P* < 0.05; ^&&^*P* < 0.01.

Hepatic fibrosis is the main pathological changes induced by *S. japonicum* infection. The liver pathological damages with the progress of liver fibrosis were also observed in our study. There was slight inflammatory cells infiltration in liver at 4 wpi. After 6 weeks infection, granulomas formation and collagen deposition were extensively accelerated (Figure 2A). Collagen deposition was mainly around the portal vein and increased rapidly thereafter (Figure 2A,B). Fibrotic response was not restricted to the area around the portal vein and had spread to normal liver tissue. But the granulomatous response and fibrosis decreased at 14 wpi. Additionally, the content of hydroxyproline kept increasing after 6 wpi and also decreased at 14 wpi (Figure 2C). The protein and mRNA levels of profibrogenic factors, including α-SMA, Collagen I, and Collagen III, were used to evaluate the activities of fibrogenesis. The expression of these fibrogenesis markers remained stable at 4 wpi compared to the control group but increased significantly at 6, 8, and 10 wpi. All of these markers decreased obviously at 14 wpi (Figure 3A,B). The mRNA level of *α-SMA*, *Collagen I*, and *Collagen III* showed similar trends (Figure 3C). These data indicated that the typical liver fibrosis of mice was induced by *S. japonicum* infection and relieved in the late stage.

### The Level of β-Actin Was Positively Correlated With Liver Fibrosis

**FIGURE 4:**
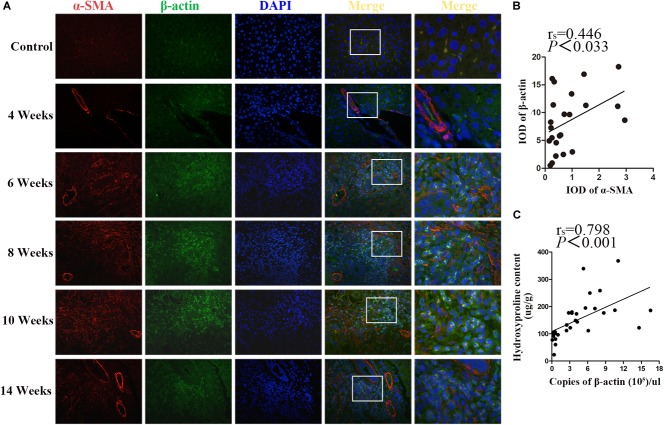
Co-localization of β-actin and α-SMA in *S. japonicum* infected mice liver. **(A)** Co-immunofluorescence staining for β-actin (green) and α-SMA (red). **(B)** The integrated optical densities (IODs) of β-actin and α-SMA were analyzed by Image Pro Plus 6.0 software. The correlation between the IOD of β-actin and α-SMA was analyzed by Spearman’s correlation analysis. **(C)** The correlation between the concentration of hepatic hydroxyproline and the level of β-actin mRNA in mice was analyzed by Spearman’s correlation analysis.

β-actin and α-SMA are the members of the actin family. It is well known that α-SMA is a dominant marker for activated HSCs during the progression of fibrotic disease. Double immunostaining for β-actin and α-SMA showed that, concomitantly with the significant increase of α-SMA upon fibrosis formation after 6 wpi infection, the expression of β-actin was also increased. Both of them peaked at 10 wpi and decreased at 14 wpi. In addition, the apparent co-localization of α-SMA and β-actin was observed in the granulomas regions (Figure 4A). As shown in Figure 4B, there was a positive correlation between α-SMA and β-actin (*r*_s_ = 0.446, *p* = 0.033). Furthermore, the concentration of hepatic hydroxyproline showing the collagen deposition in liver was determined. Besides, the correlation between the concentration of hydroxyproline and the mRNA level of β-actin was analyzed. As shown in Figure 4C, a significantly positive correlation was observed between them (*r*_s_ = 0.798, *P* < 0.001). Thus, our results suggested that β-actin was positively correlated with the severity of fibrosis.

## Discussion

In *S. japonicum* infection, recruitment and activation of HSCs in granulomas are the key elements contributing to hepatic fibrosis formation in the liver (Chuah et al., 2014;Carson et al., 2018). β-actin, a highly conserved protein used as an internal control in biological studies, shares more than 93% sequence identity with α-SMA. It is well known that α-SMA is a hallmark of activated HSCs and increases in the liver during *S. japonicum* infection (Bartley et al., 2006; Wen et al., 2017). Hence, it is vital to clarify whether β-actin is stably expressed and fit for application as an internal control upon hepatic fibrosis formation. The data in the present study indicated that accompanied with the progress of fibrotic response in *S. japonicum* infected mouse liver, the gene and protein expression levels of β-actin changed and significantly correlated with the severity of liver fibrosis in a mouse model.

Ideal housekeeping genes or proteins used as internal controls must be expressed constitutively, ubiquitously, and uniformly in cells and tissue, and are not likely to be regulated by any experimental treatment (Tunbridge et al., 2011; Lv et al., 2015). Some recent studies reported that the expressions of several typical internal controls were not stable with the stimulation by pharmacological agents or under special physiological and pathological conditions. For example, β-actin, GAPDH, and peptidylprolyl isomerase A (PPIA) showed significant fluctuations in neural stem cells under hypoxic conditions (Yao et al., 2012). The expression level of PPIA increased in cortical neuronal cultures following pretreatment with oxidative or ischemic injury. Moreover, in hypoxic human chondrocytes, the expressions of β-actin and GAPDH were unstable (Foldager et al., 2009). GAPDH expression was also observed to vary widely in cells under insulin or mitogen stimulation (Nasrin et al., 1990; Rao et al., 1990). In our study, we found that β-actin slightly increased at 4 wpi. With dramatic fibrosis formation after 6 wpi, both the gene and protein levels of β-actin increased sharply. This increase is consistent with the gene expression pattern of β-actin in the transcriptome data (NCBI SRA database: SRP073956) of mice infected with *S. mansoni* (Wijayawardena et al., 2016). However, other two typical internal controls of GAPDH and β-Tubulin remained stable during *S. japonicum* infection. In addition, β-actin increased in fibrotic liver from both female and male infected mice, and there is no difference in both them (Supplementary Figure S1). The elevated expression of β-actin may be due to that β-actin, a major component of the cytoskeleton protein (Durham and Herman, 2009; Chen et al., 2017), has the function to meet the need for tissue repair via filament remodeling. Thus, β-actin is not an appropriate internal control to normalize data in hepatic fibrosis with *schistosome* infection, as its variable expression level may lead to inaccurate calculation of other genes.

The dynamic change of β-actin was similar with the profibrogenic factors, including α-SMA, Collagen I, and Collagen III. Spearman’s correlation analysis also revealed a positive correlation between the concentration of hydroxyproline and the level of β-actin mRNA in liver. Therefore, the expression level of β-actin might be an indicator of the severity of fibrosis. It is well known that fibrosis is the final outcome of several chronic liver diseases. It has various etiologies, including viral infections, drug stimulation, toxic damage, metabolic, and genetic diseases (Campana and Iredale, 2017). The expression level of β-actin in fibrotic liver induced by CCl4 was detected by western blot. Interestingly, we found that it also increased in the liver stimulated with CCl4 (Supplementary Figure S2), but GAPDH and Tubulin remained stable. These data were similar to the results in schistosomiasis mice, which implied that β-actin might be variable in general hepatic fibrosis models. This speculation is worthwhile to be validated in more fibrotic models.

## Conclusion

This study clarified that the expression level of liver β-actin increased with the progression of fibrosis progression, revealing that β-actin is not a suitable internal control in hepatic fibrosis associated with schistosomiasis. Our study provides experimental evidence for selecting suitable internal control while detecting target genes or proteins in the study of schistosomiasis.

## Author Contributions

BZ, JL, QS, and LW performed the experiments. XW, BZ, JL, and LS collected the patients serum in epidemic area. BZ, XW, and LS contributed to the data analysis. ZW and DY designed the study. ZW, DY, and BZ wrote the manuscript. All authors revised and approved the manuscript.

## Conflict of Interest Statement

The authors declare that the research was conducted in the absence of any commercial or financial relationships that could be construed as a potential conflict of interest.

## References

[B1] Akpolat N. Yahsi S. Godekmerdan A. Yalniz M. Demirbag K. (2005). The value of alpha-SMA in the evaluation of hepatic fibrosis severity in hepatitis B infection and cirrhosis development: a histopathological and immunohistochemical study. *Histopathology* 47 276–280. 10.1111/j.1365-2559.2005.02226.x 16115228

[B2] Barsoum R. S. Esmat G. El-Baz T. (2013). Human schistosomiasis: clinical perspective: review. *J. Adv. Res.* 4 433–444. 10.1016/j.jare.2013.01.005 25685450 PMC4293888

[B3] Bartley P. B. Ramm G. A. Jones M. K. Ruddell R. G. Li Y. McManus D. P. (2006). A contributory role for activated hepatic stellate cells in the dynamics of Schistosoma japonicum egg-induced fibrosis. *Int. J. Parasitol.* 36 993–1001. 10.1016/j.ijpara.2006.04.015 16806222

[B4] Campana L. Iredale J. P. (2017). Regression of liver fibrosis. *Semin. Liver Dis.* 37 1–10. 10.1055/s-0036-1597816 28201843

[B5] Carson J. P. Ramm G. A. Robinson M. W. McManus D. P. Gobert G. N. (2018). Schistosome-induced fibrotic disease: the role of hepatic stellate cells. *Trends Parasitol.* 34 524–540. 10.1016/j.pt.2018.02.005 29526403

[B6] Chen X. Zheng J. Cai J. Li H. Li S. Wang L. (2017). The cytoskeleton protein beta-actin may mediate T cell apoptosis during acute rejection reaction after liver transplantation in a rat model. *Am. J. Transl. Res.* 9 4888–4901. 29218087 PMC5714773

[B7] Chuah C. Jones M. K. Burke M. L. McManus D. P. Gobert G. N. (2014). Cellular and chemokine-mediated regulation in schistosome-induced hepatic pathology. *Trends Parasitol.* 30 141–150. 10.1016/j.pt.2013.12.009 24433721

[B8] Colley D. G. Bustinduy A. L. Secor W. E. King C. H. (2014). Human schistosomiasis. *Lancet* 383 2253–2264. 10.1016/s0140-6736(13)61949-2 24698483 PMC4672382

[B9] Durham J. T. Herman I. M. (2009). Inhibition of angiogenesis in vitro: a central role for beta-actin dependent cytoskeletal remodeling. *Microvasc. Res.* 77 281–288. 10.1016/j.mvr.2008.12.003 19323981 PMC2706787

[B10] Foldager C. B. Munir S. Ulrik-Vinther M. Soballe K. Bunger C. Lind M. (2009). Validation of suitable house keeping genes for hypoxia-cultured human chondrocytes. *BMC Mol. Biol.* 10:94. 10.1186/1471-2199-10-94 19818117 PMC2764705

[B11] Kuramitsu K. Sverdlov D. Y. Liu S. B. Csizmadia E. Burkly L. Schuppan D. (2013). Failure of fibrotic liver regeneration in mice is linked to a severe fibrogenic response driven by hepatic progenitor cell activation. *Am. J. Pathol.* 183 182–194. 10.1016/j.ajpath.2013.03.018 23680654 PMC3702745

[B12] Li-Juan Z. Zhi-Min X. Ying-Jun Q. Hui D. Shan L. Jing X. (2017). [Endemic status of schistosomiasis in People’s Republic of China in 2016]. *Zhongguo Xue Xi Chong Bing Fang Zhi Za Zhi* 29 669–677. 10.16250/j.32.1374.2017204 29469441

[B13] Lv W. Zheng J. Luan M. Shi M. Zhu H. Zhang M. (2015). Comparing the evolutionary conservation between human essential genes, human orthologs of mouse essential genes and human housekeeping genes. *Brief Bioinform.* 16 922–931. 10.1093/bib/bbv025 25911641

[B14] MacQueen A. J. Baggett J. J. Perumov N. Bauer R. A. Januszewski T. Schriefer L. (2005). ACT-5 is an essential Caenorhabditis elegans actin required for intestinal microvilli formation. *Mol. Biol. Cell* 16 3247–3259. 10.1091/mbc.E04-12-1061 15872090 PMC1165408

[B15] Nasrin N. Ercolani L. Denaro M. Kong X. F. Kang I. Alexander M. (1990). An insulin response element in the glyceraldehyde-3-phosphate dehydrogenase gene binds a nuclear protein induced by insulin in cultured cells and by nutritional manipulations in vivo. *Proc. Natl. Acad. Sci. U.S.A.* 87 5273–5277. 10.1073/pnas.87.14.5273 2164673 PMC54305

[B16] Peng H. Zhang Q. Li X. Liu Z. Shen J. Sun R. (2016). IL-33 contributes to schistosoma japonicum-induced hepatic pathology through induction of M2 macrophages. *Sci. Rep.* 6:29844. 10.1038/srep29844 27445267 PMC4956744

[B17] Rao G. N. Sardet C. Pouyssegur J. Berk B. C. (1990). Differential regulation of Na+/H+ antiporter gene expression in vascular smooth muscle cells by hypertrophic and hyperplastic stimuli. *J. Biol. Chem.* 265 19393–19396. 2174035

[B18] Ruan W. Lai M. (2007). Actin, a reliable marker of internal control? *Clin. Chim. Acta* 385 1–5. 10.1016/j.cca.2007.07.003 17698053

[B19] Song L. Wu X. Ning A. Wu Z. (2017). Lessons from a 15-year-old boy with advanced schistosomiasis japonica in China: a case report. *Parasitol. Res.* 116 1787–1791. 10.1007/s00436-017-5473-3 28508167

[B20] Sturzenbaum S. R. Kille P. (2001). Control genes in quantitative molecular biological techniques: the variability of invariance. *Comp. Biochem. Physiol. B Biochem. Mol. Biol.* 130 281–289. 10.1016/S1096-4959(01)00440-7 11567890

[B21] Thomas J. A. Pope C. Wojtacha D. Robson A. J. Gordon-Walker T. T. Hartland S. (2011). Macrophage therapy for murine liver fibrosis recruits host effector cells improving fibrosis, regeneration, and function. *Hepatology* 53 2003–2015. 10.1002/hep.24315 21433043

[B22] Tunbridge E. M. Eastwood S. L. Harrison P. J. (2011). Changed relative to what? Housekeeping genes and normalization strategies in human brain gene expression studies. *Biol. Psychiatry* 69 173–179. 10.1016/j.biopsych.2010.05.023 20673871

[B23] Wen Z. Ji X. Tang J. Lin G. Xiao L. Liang C. (2017). Positive feedback regulation between transglutaminase 2 and toll-like receptor 4 signaling in hepatic stellate cells correlates with liver fibrosis post *Schistosoma japonicum* infection. *Front. Immunol.* 8:1808. 10.3389/fimmu.2017.01808 29321784 PMC5733538

[B24] Wijayawardena B. K. Minchella D. J. DeWoody J. A. (2016). The influence of trematode parasite burden on gene expression in a mammalian host. *BMC Genomics* 17:600. 10.1186/s12864-016-2950-5 27514777 PMC4982272

[B25] Yao L. Chen X. Tian Y. Lu H. Zhang P. Shi Q. (2012). Selection of housekeeping genes for normalization of RT-PCR in hypoxic neural stem cells of rat in vitro. *Mol. Biol. Rep.* 39 569–576. 10.1007/s11033-011-0772-8 21633896

