# Supplementary material for: β-Actin: Not a Suitable Internal Control of Hepatic Fibrosis Caused by Schistosoma japonicum
Source: Front Microbiol. 2019 Jan 31;10:66. doi: 10.3389/fmicb.2019.00066 (PMC6365423; doi:10.3389/fmicb.2019.00066)
Supplement: Supplementary file 1 [file Presentation_1.PPTX]

## Slide 1
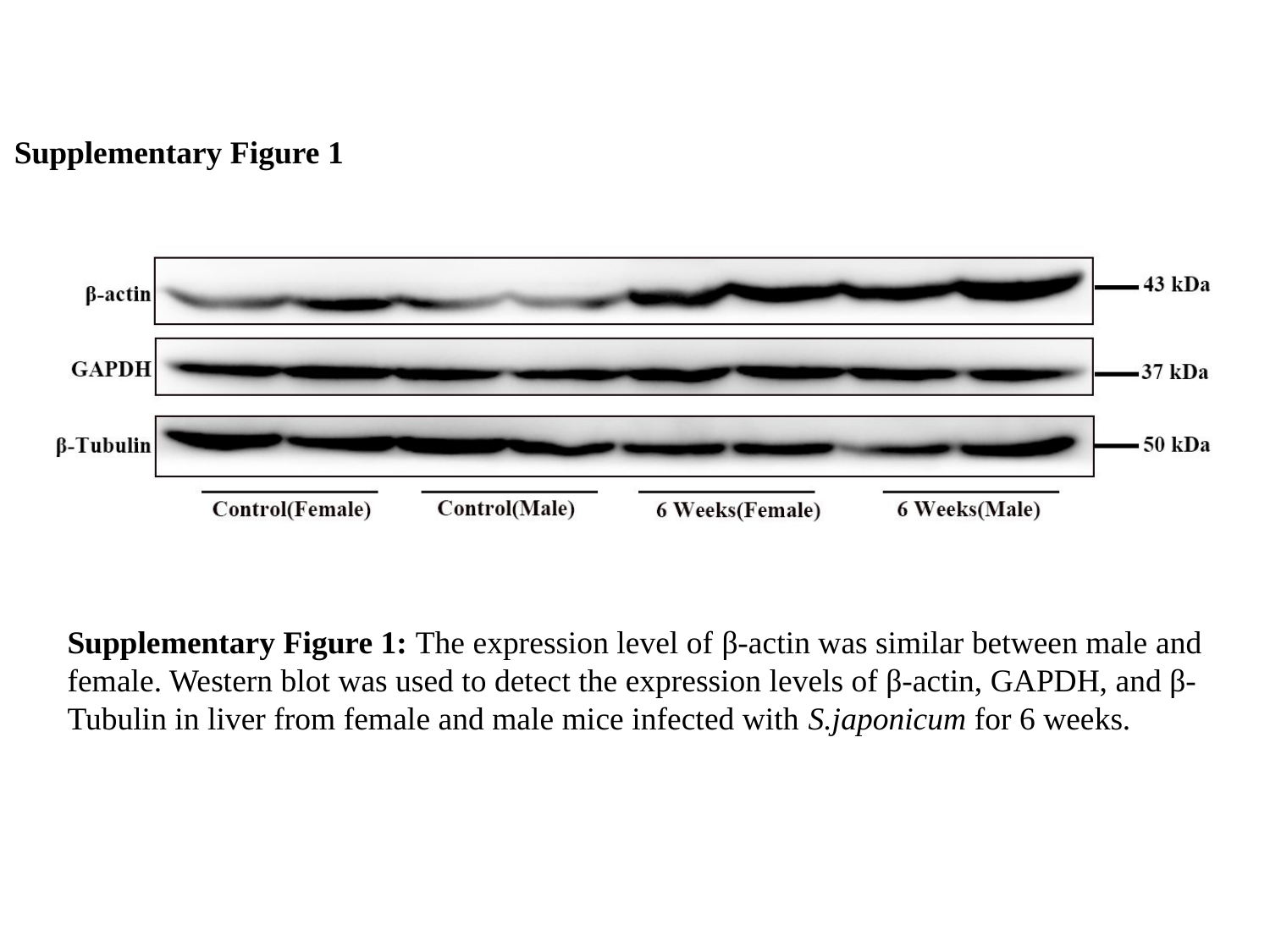

Supplementary Figure 1
Supplementary Figure 1: The expression level of β-actin was similar between male and female. Western blot was used to detect the expression levels of β-actin, GAPDH, and β-Tubulin in liver from female and male mice infected with S.japonicum for 6 weeks.

## Slide 2
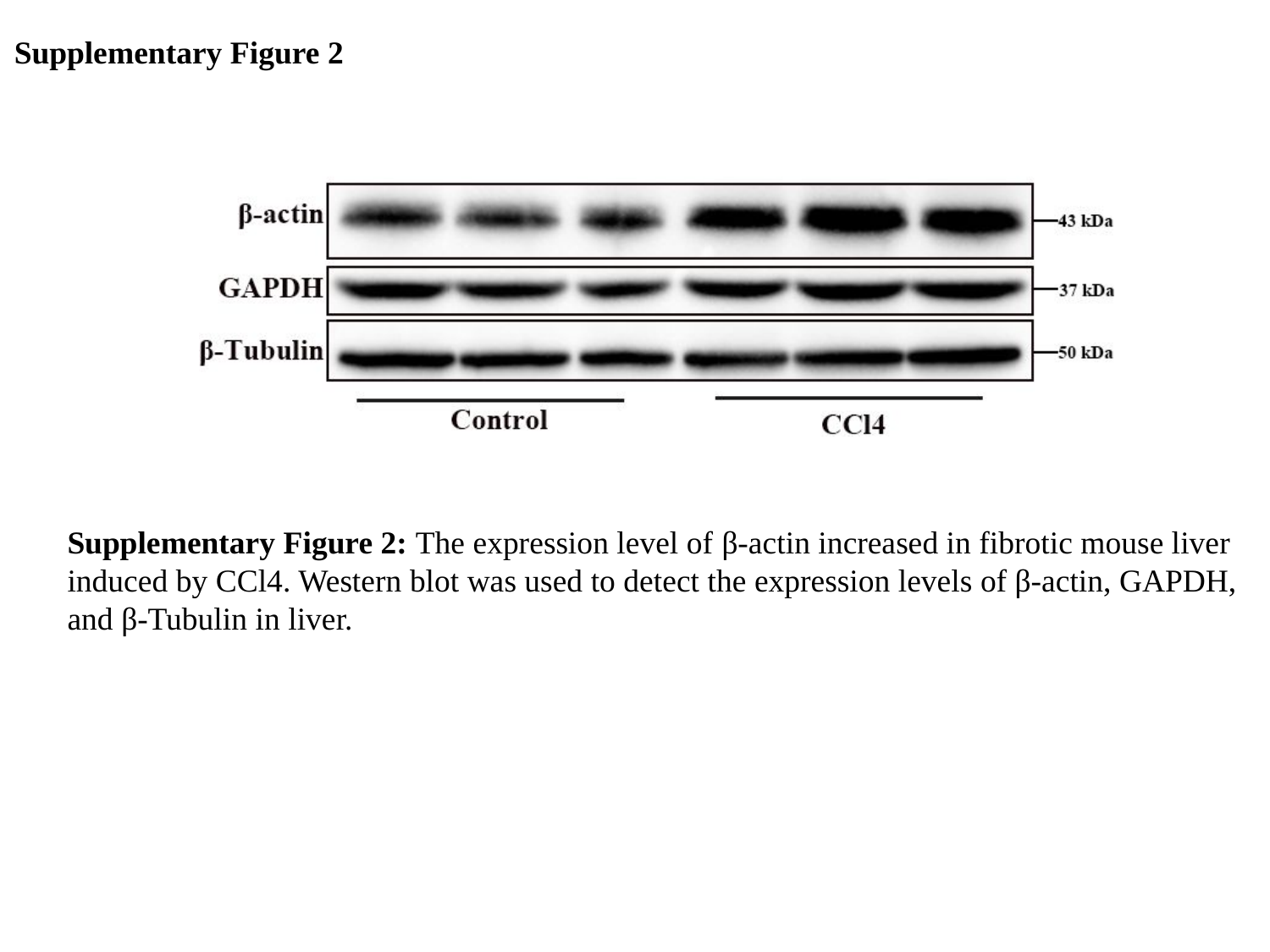

Supplementary Figure 2
Supplementary Figure 2: The expression level of β-actin increased in fibrotic mouse liver induced by CCl4. Western blot was used to detect the expression levels of β-actin, GAPDH, and β-Tubulin in liver.
